# Finite Element Analysis on Nanomechanical Detection of Small Particles: Toward Virus Detection

**DOI:** 10.3389/fmicb.2016.00488

**Published:** 2016-04-14

**Authors:** Gaku Imamura, Kota Shiba, Genki Yoshikawa

**Affiliations:** ^1^World Premier International Research Center Initiative, International Center for Materials Nanoarchitectonics, National Institute for Materials ScienceTsukuba, Japan; ^2^International Center for Young Scientists, National Institute for Materials ScienceTsukuba, Japan

**Keywords:** nanomechanical sensors, cantilever sensors, membrane-type surface stress sensors (MSS), finite element analysis (FEA), virus detection

## Abstract

Detection of small particles, including viruses and particulate matter (PM), has been attracting much attention in light of increasing need for environmental monitoring. Owing to their high versatility, a nanomechanical sensor is one of the most promising sensors which can be adapted to various monitoring systems. In this study, we present an optimization strategy to efficiently detect small particles with nanomechanical sensors. Adsorption of particles on the receptor layer of nanomechanical sensors and the resultant signal are analyzed using finite element analysis (FEA). We investigate the effect of structural parameters (e.g., adsorption position and embedded depth of a particle and thickness of the receptor layer) and elastic properties of the receptor layer (e.g., Young's modulus and Poisson's ratio) on the sensitivity. It is found that a membrane-type surface stress sensors (MSS) has the potential for robust detection of small particles.

## Introduction

Nanomechanical sensors have been attracting great attention because of their versatility. For example, they can detect diverse chemical species ranging from gaseous to biological molecules, including volatile organic compounds (VOCs), DNA, and proteins (Barnes et al., 1994; Gimzewski et al., 1994; Thundat et al., 1994; Buchapudi et al., 2011). Detection of bioorganisms such as viruses and bacteria has also been reported (Buchapudi et al., 2011). Since the first demonstration of nanomechanical sensing in 1994 (Gimzewski et al., 1994), cantilever-type sensors with optical read-outs have been widely used. In 2011, a membrane-type surface stress sensor (MSS) was developed and achieved both high sensitivity and small size by means of structural optimization with chip-integrated piezoresistive read-out (Yoshikawa et al., 2011). This MSS platform also demonstrated the detection of proteins using a simple dipping system that is compatible with a standard 96-well plate for practical assays (Hosokawa et al., 2014).

Owing to these advantages, nanomechanical sensors are expected to be a key technology to environmental monitoring systems. In addition to toxic chemical species in air, there is a great need for detection of airborne particles including dust, particulate matter (PM), and viruses (Aliabadi et al., 2011; Després et al., 2012; Nemmar et al., 2013; Kim et al., 2015). As nanomechanical sensors can recognize such particles by detecting mechanical properties, it is important to understand the mechanics of the nanomechanical sensing system: Adsorption of particles on the receptor layer and the stress and deformation caused by adsorbed particles.

In the present study, we investigate the nanomechanical detection of particles using finite element analysis (FEA). We focus on the two types of nanomechanical sensors; cantilever-type sensors and MSS, and explore an optimized structure for the efficient detection of particles through the mechanical stress induced in the receptor layer. We also discuss the perspective on virus detection using these nanomechanical sensors.

## Materials and methods

Analytical solutions of nanomechanical sensing are available for a simple cantilever model. For example, the deflection of the free-end of a cantilever (Δ*z*) induced by isotropic internal strain in a receptor layer (ε_*f*_) is given by the following equation (Yoshikawa, 2011):

(1) $$\begin{array}{l} \Delta z=\frac{3l^{2}\left(t_{f}\;+\;t_{c}\right)}{\left(A\;+\;4\right)t_{f}^{2}+\left(A^{-1}\;+\;4\right)t_{c}^{2}\;+\;6t_{f}t_{c}}\varepsilon_{f}, \end{array}$$

(2) $$\begin{array}{l} A=\frac{E_{f}w_{f}t_{f}\left(1-\nu_{c}\right)}{E_{c}w_{c}t_{c}\left(1-\nu_{f}\right)}, \end{array}$$

where *l* is the length of a cantilever, and *E*_*f*_ (*E*_*c*_), ν_*f*_ (ν_*c*_), and *t*_*f*_ (*t*_*c*_) are the Young's modulus, the Poisson's ratio, and the thickness of a receptor layer (a cantilever), and *w*_*c*_ and *w*_*f*_ are the width of a cantilever and a receptor layer, respectively. If a cantilever is covered with a very thin receptor layer (*t*_*c*_≫ *t*_*f*_ and *w*_*c*_ = *w*_*f*_), Equation (1) reduces to the following equation, which is known as the Stoney's equation (Stoney, 1909):

(3) $$\begin{array}{l} \Delta z=\frac{3\left(1-\nu_{c}\right)l^{2}}{E_{c}t_{c}^{2}}\sigma_{\text{surf}}, \end{array}$$

where σ_surf_ is the surface stress defined as σ_surf_ = σ_*f*_ · *t*_*f*_, and σ_*f*_ = ε_*f*_ · *E*_*f*_/(1 − ν_*f*_). However, the application of these models is limited to analytically simple problems. To investigate more complex systems, FEA is an effective option, providing numerical solutions. Since the adsorption of particles on a solid receptor layer with three dimensional stress distribution is too complicated to be analytically modeled, we employed FEA in the present study.

**Figure 1:**
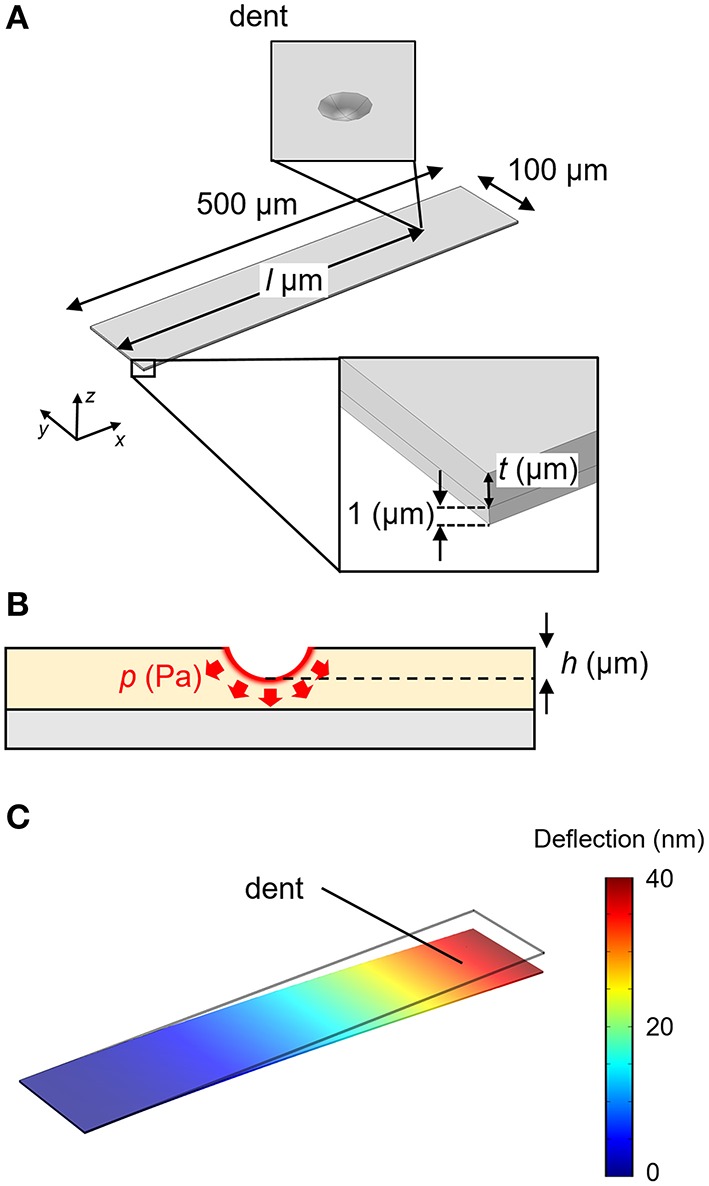
**(A)** Structure of the cantilever-type sensor. **(B)** Cross sectional image of the cantilever at the dent. **(C)** Result of the FEA simulation for the cantilever-type sensor. The distribution of displacement is plotted as a color gradient.

**Figure 2:**
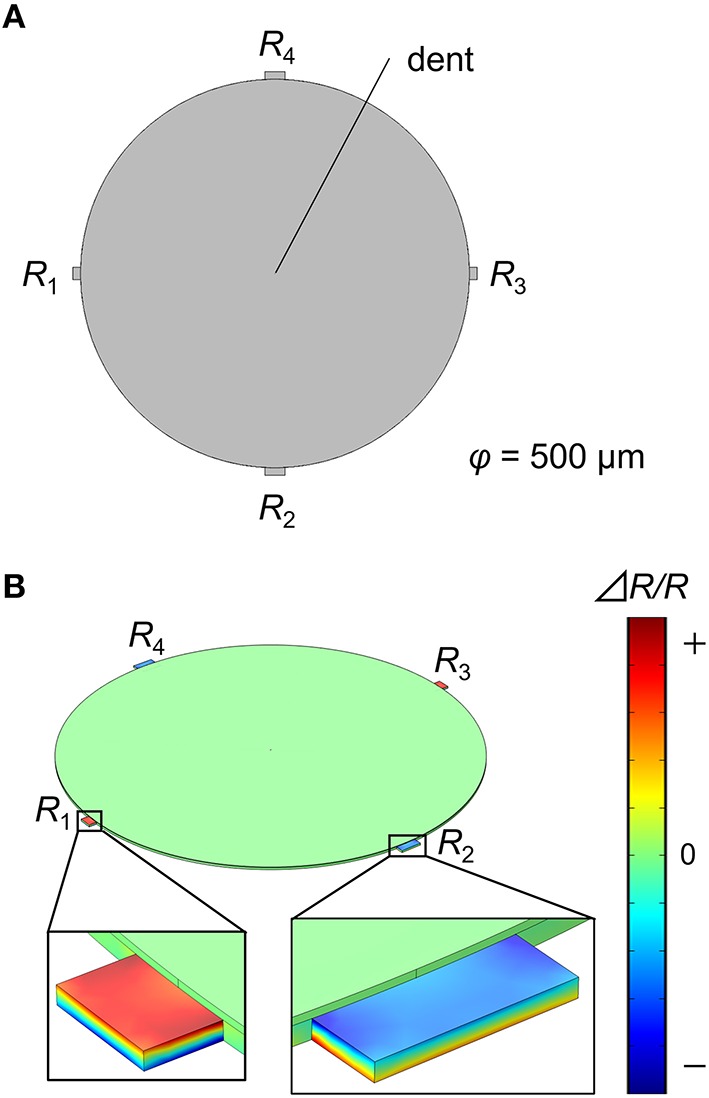
**(A)** Structure of the MSS. **(B)** Result of the FEA simulation for the MSS. The distribution of relative resistance change is plotted as a color gradient.

FEA simulations were performed in COMSOL Multiphysics 5.1® with the Structural Mechanics module. Each structure was meshed with 20,000~60,000 elements, which give sufficient resolution for the present simulations. We investigated the deflection of a silicon cantilever with dimensions 500 × 100 × 1 μm as shown in Figure 1A. A fixed constraint was applied on one end (fixed-end). The receptor layer with a thickness of *t* μm is coated on the cantilever. A particle with a radius of 0.1 μm is embedded in the receptor layer with a depth of *h* μm, applying a constant pressure *p* Pa at the interface (Figure 1B). To simulate such a situation, we set a dent on the receptor layer with a boundary load of *p* Pa at the surface. The dent is located at *l* μm from the fixed-end. We investigated the deflection; the displacement of the free-end of the cantilever (Figure 1C). In the case of MSS, the diameter and the thickness of the membrane are set at 500 and 2.5 μm, respectively, with a *t*-μm-thick receptor layer on it (Figure 2A). The dent is also placed on the receptor layer (default position is set at the center) to simulate the adsorption of a particle. We investigated the change in the relative resistance (Δ*R*_tot_*/R*_tot_) of a full Wheatstone bridge in an MSS composed of four resistors embedded in the sensing beams, *R*_1_–*R*_4_ (Figure 2B). The sensing signal, *V*_out_ is approximately described as:

(4) $$\begin{array}{l} V_{\text{out}}=\frac{V_{B}}{4}\left(\frac{\Delta R_{1}}{R_{1}}-\frac{\Delta R_{2}}{R_{2}}+\frac{\Delta R_{3}}{R_{3}}-\frac{\Delta R_{4}}{R_{4}}\right)=\frac{V_{B}}{4}\left(\frac{\Delta R_{\text{tot}}}{R_{\text{tot}}}\right), \end{array}$$

where *V*_*B*_ is the bridge voltage, andΔ*R*_*i*_ is the change in the resistance of *R*_*i*_ (Yoshikawa et al., 2011). A fixed constraint was applied on the end of each beam.

## Results

### Cantilever-type sensor

**Figure 3:**
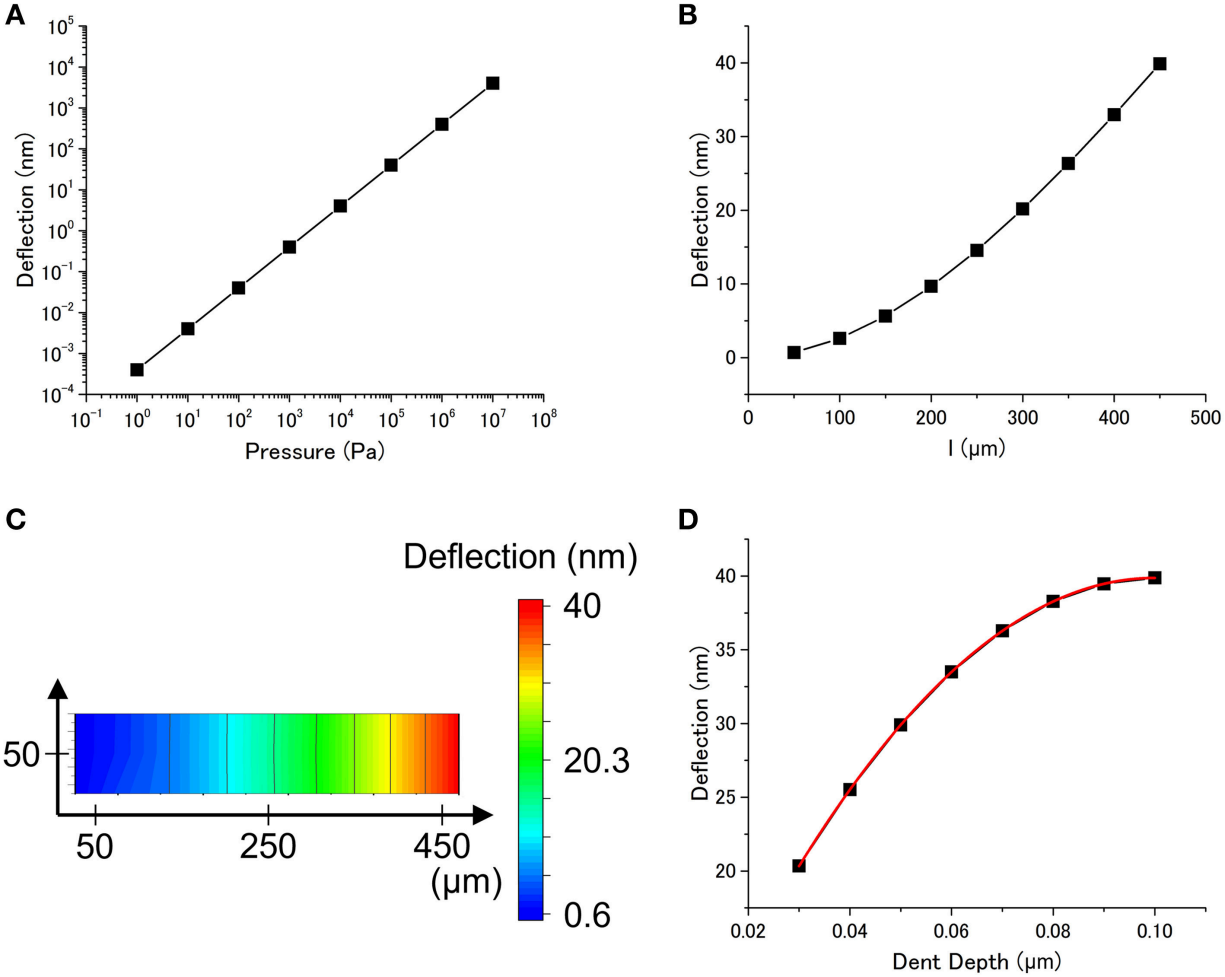
**(A)** Dependence of the deflection on the applied pressure (*l* = 450 μm, *h* = 0.1 μm, *t* = 1 μm, *E* = 1 × 10^9^ Pa, ν = 0.4). **(B)** Dependence of the deflection on the position of the dent (distance from the fixed end). **(C)** Dependence of the deflection on the position of the dent. The resultant cantilever deflection is plotted as a color gradient (*p* = 1 × 10^5^ Pa, *h* = 0.1 μm, *t* = 1 μm, *E* = 1 × 10^9^ Pa, ν = 0.4). **(D)** Dependence of the deflection on the dent depth (*p* = 1 × 10^5^ Pa, *l* = 450 μm, *t* = 1 μm, *E* = 1 × 10^9^ Pa, ν = 0.4). The plot is fitted with a quadratic function (red curve).

The dependence on the applied pressure, *p*, is investigated. It is clear that the deflection of the cantilever is exactly proportional to *p* over a wide range (Figure 3A). This result suggests that the dependence of the deflection on the other parameters is not affected by *p*. We confirmed this condition by investigating the dependence on the other parameters while varying *p*. Figures 3B,C show the dependence on *l*, showing a natural consequence of simple mechanics: a particle adsorbed near the end of the cantilever leads to a larger deflection. The depth of the dent which corresponds to the embedded depth of a particle strongly affects the deflection. Figure 3D shows the deflection as a function of dent depth *h* with a nonlinear relationship. Since the data in Figure 3D can be fitted well with the quadratic function, the deflection is found to be related with the projected area of the dent *s* [*s* = π (2*rh–h*^2^), where *r* is a radius of a particle], rather than the surface area of the dent *S* (*S* = 2π*rh*).

**Figure 4:**
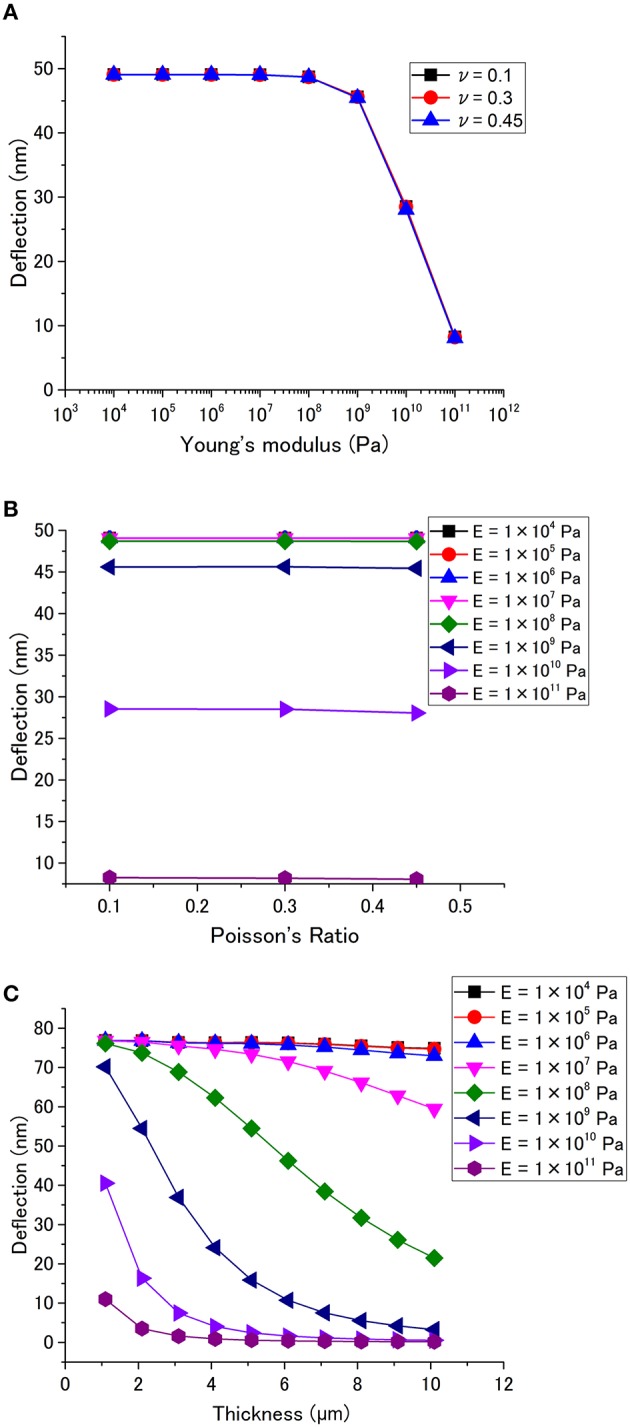
**Cantilever deflection as a function of (A) Young's modulus, and (B) Poisson's ratio**. (*p* = 1 × 10^5^ Pa, *l* = 450 μm, *h* = 0.1 μm, *t* = 1 μm) **(C)** Dependence of the deflection on the thickness for different Young's modulus (*p* = 1 × 10^5^ Pa, *l* = 450 μm, *h* = 0.1 μm).

Figures 4A,B show the effects of Young's modulus *E* and Poisson's ratio ν, respectively. It has been found that the deflection is independent of ν, while *E* significantly affects the deflection. The deflection exhibits little dependence on *E* below 10^8^ Pa. However, a higher Young's modulus yields a lower deflection when *E* is larger than 10^9^ Pa. The thickness dependence is summarized in Figure 4C. It clearly shows that the thickness dependence is significantly affected by Young's modulus. The deflection decreases with increasing thickness, and it becomes drastic with *E* > 10^7^ Pa.

### MSS

**Figure 5:**
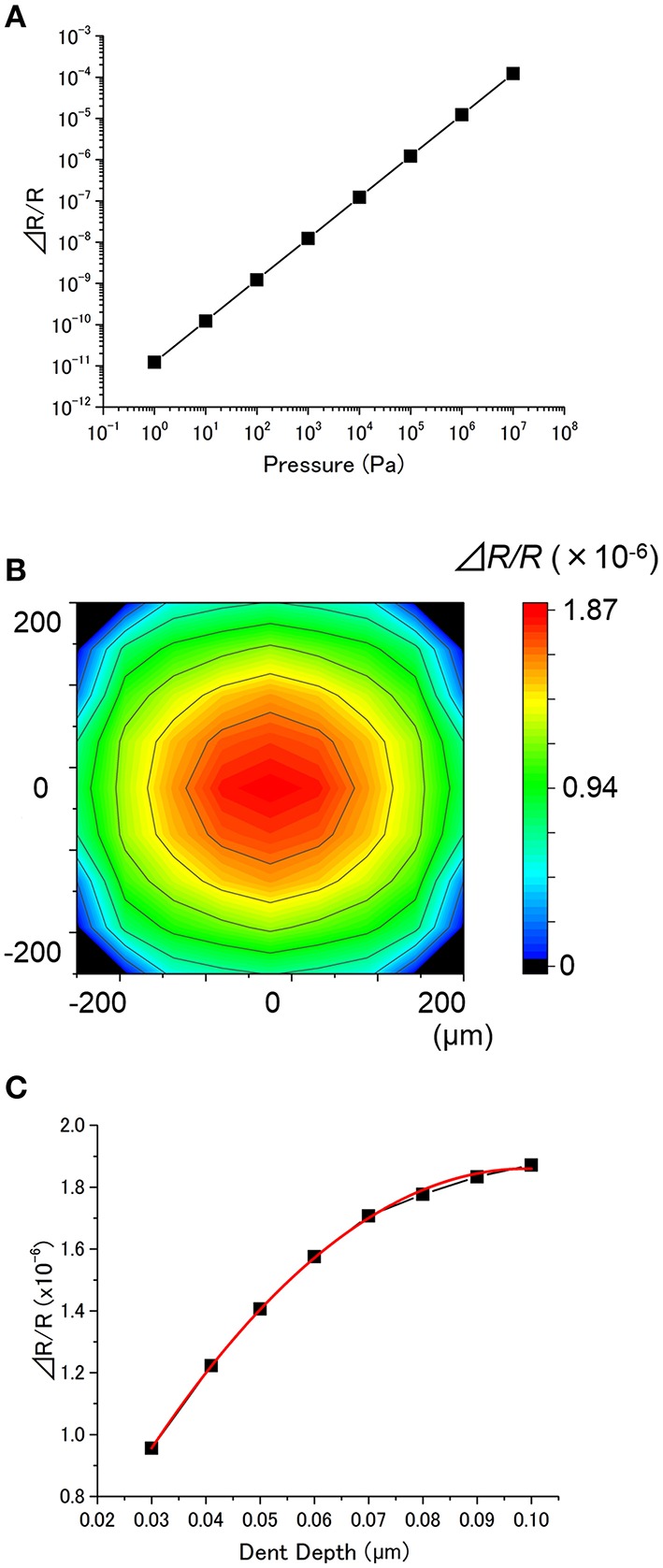
**(A)** Dependence of Δ*R/R* on the applied pressure (the dent is at the center, *h* = 0.1 μm, *t* = 1 μm, *E* = 1 × 10^9^ Pa, ν = 0.4). **(B)** Dependence of Δ*R/R* on the position of the dent. The resultant relative resistance change is plotted as a color gradient (*p* = 1 × 10^5^ Pa, *h* = 0.1 μm, *t* = 1 μm, *E* = 1 × 10^9^ Pa, ν = 0.4). **(C)** Dependence of Δ*R/R* on the dent depth (*p* = 1 × 10^5^ Pa, the dent is at the center, *t* = 1 μm, *E* = 1 × 10^9^ Pa, ν = 0.4). The plot is fitted with a quadratic function (red curve).

The effects of applied pressure *p*, position, and dent depth *h* on Δ*R*_tot_*/R*_tot_ were investigated for MSS. Figure 5A depicts Δ*R*_tot_*/R*_tot_ as a function of *p*, showing a linear relationship over a wide range. Same as a cantilever-type sensor, variation in *p* is found not to affect the dependence on other parameters in the case of MSS as well. Figure 5B shows the dependence on the position of the dent. It has been found that a higher signal can be obtained when a particle adsorbs near the center of the membrane. An interesting feature of MSS is that it is more robust in position of the dent compared to cantilever-type sensors. In the case of cantilever-type sensors, a dent at *l* = 450 μm causes ~60 times larger deflection than a dent at *l* = 50 μm. On the other hand, the signal caused by a dent at the center of MSS is not 10 times larger than the signal caused by a dent at 50 μm from the edge. This result indicates that MSS is more robust in the adsorption position of a particle compared to cantilever-type sensors. The dependence of Δ*R*_tot_*/R*_tot_ on a dent depth *h* is quite similar to the case of cantilever-type sensors; a higher sensing signal can be obtained for a deeper dent (Figure 5C).

**Figure 6:**
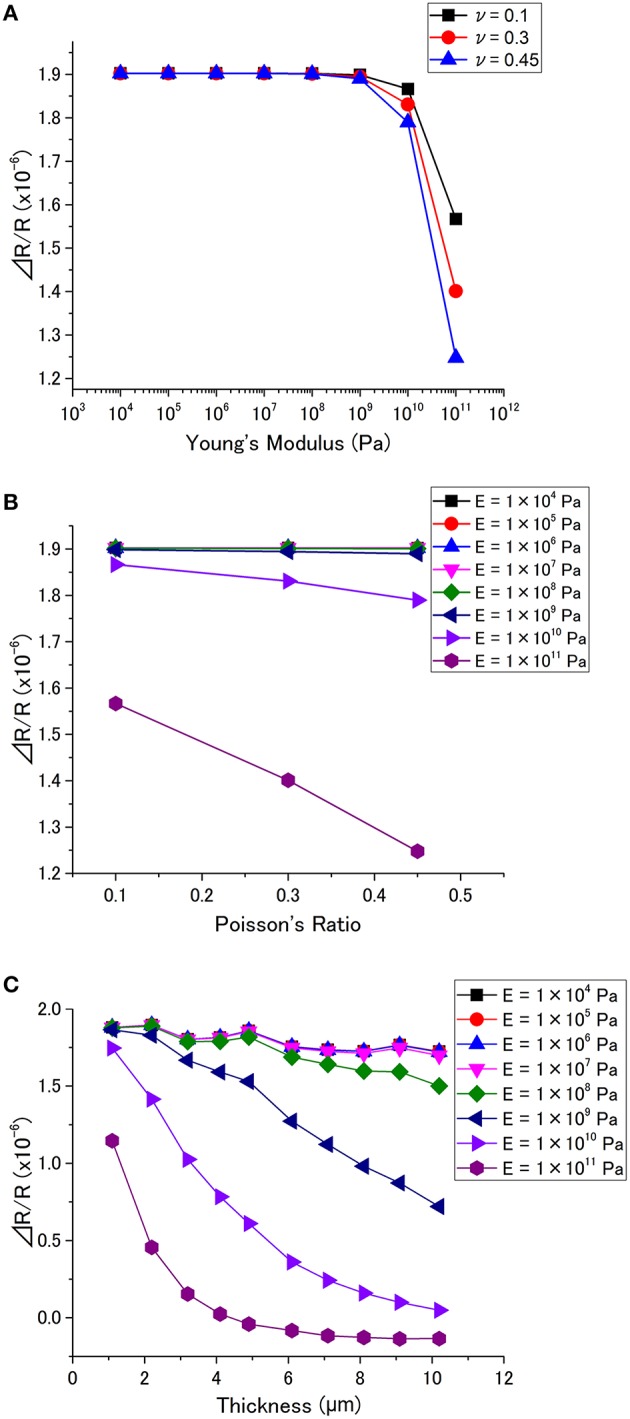
**ΔR/R as a function of (A) Young's modulus, and (B) Poisson's ratio (*p* = 1 × 10^5^ Pa, the dent is at the center, *h* = 0.1 μm, *t* = 1 μm)**. **(C)** Dependence of Δ*R/R* on the thickness for different Young's modulus (*p* = 1 × 10^5^ Pa, the dent is at the center, *h* = 0.1 μm).

Δ*R*_tot_*/R*_tot_ for various *E* and ν are plotted in Figure 6. Figure 6A shows Δ*R*_tot_*/R*_tot_ has little dependence on *E* below 10^9^ Pa, while Δ*R*_tot_*/R*_tot_ decreases with *E* over 10^9^ Pa. As shown in Figure 6B, Poisson's ratio does not significantly affect Δ*R*_tot_*/R*_tot_ when *E* is below 10^9^ Pa. However, a higher Poisson's ratio results in a lower Δ*R*_tot_*/R*_tot_ in the case of *E* > 10^9^ Pa. The simulated Δ*R*_tot_*/R*_tot_ as a function of thickness is plotted in Figure 6C. The behavior is similar to the case of cantilever-type sensors (Figure 4C). The receptor layer with a soft material (*E* < 10^8^ Pa) exhibits little dependence on thickness, while the signal drastically decreases with thickness for a hard material (*E* > 10^8^ Pa).

## Discussion

The presented results indicate that a thinner and softer receptor layer will lead to a larger signal for the purpose of the detection of particles. It is known that there is an optimum thickness which yields maximum deflection in the cases of two typical nanomechanical sensing systems: the 2D stress applied homogeneously on the surface of a receptor layer, and the isotropic internal strain induced in a receptor layer (Yoshikawa, 2011; Yoshikawa et al., 2014). In contrast to such systems, an optimum thickness has not been found for any Young's moduli in the present simulation. This difference can be ascribed to the lack of the lateral stress enhancement effect in the case of particle adsorption. The effective stress induced on a sensing body is enhanced with increasing thickness of a receptor layer for the 2D stress or the isotropic internal strain because the lateral component of the force is applied further away from the neutral axis of the system. However, such effect for the present simulation is marginal because an effective force in the lateral direction is minute, hardly contributing to the deflection. Thus, the stiffening effect caused by the increase in thickness and/or Young's modulus becomes dominant, resulting in a smaller signal for thicker and stiffer receptor layer. The dependence on the position is found to be consistent with simple mechanics; a larger distance from a fixed-end provides a larger deflection in the cases of both cantilever-type and MSS.

### Toward virus detection

Based on these results, here we discuss a strategy to efficiently detect viruses with nanomechanical sensors. Several studies reported virus detection using nanomechanical sensors. These studies fall into two categories in terms of the operation mode: dynamic mode and static mode. Nanomechanical sensors operated in dynamic mode detect viruses by their mass (more specifically, shifts in the resonant frequency; Johnson et al., 2006; Braun et al., 2009; Cha et al., 2009; Capobianco et al., 2010) while static mode operates based on the stress or strain (Gunter et al., 2003; Sreepriya and Hai-Feng, 2006; Alodhayb et al., 2013; Xu et al., 2014; Gorelkin et al., 2015; Kim et al., 2015). Thus, the FEA simulation in the present study can be adapted to the detection of viruses in static mode.

Previous studies revealed that the force caused by a virus attached to a cell is on the order of 10 pN (Lee et al., 2006; Sieben et al., 2012; Tsai et al., 2015). Assuming that a virus of 0.1 μm in radius is embedded 0.1 μm deep into a cell, the pressure applied to the interface is estimated to be ~10^3^ Pa. In the present simulations, the applied pressure *p* is set at 1 × 10^5^ Pa (Figures 3B–D, 4, 5B,C, 6), which is two orders of magnitude higher than the pressure caused by a virus. As the deflection and relative resistance changes of a cantilever-type sensor and MSS, respectively, are proportional to the applied pressure *p*, it is expected that the deflection/relative resistance change increases linearly with the number of adsorbed viruses on the receptor layer if the adsorbed positions of viruses are effectively regarded as constant. Accordingly, the presented simulations, in which *p* is set at 1 × 10^5^ Pa, are comparable to a system where a hundred of viruses are attached to a specific position on the receptor layer. Considering that the typical detection limits for cantilever-type sensors and MSS are estimated as ~1 nm and ~10^−6^, respectively (Yoshikawa et al., 2011, 2012), a detectable signal can be obtained when ~10^2^ of viruses adsorb near the free-end of a cantilever or the center of MSS with a receptor layer having optimal properties (*t* = 1 μm, *h* = 0.1 μm, *E* < 10^8^ Pa). To compare with typical nanomechanical sensing based on isotropic internal strain or surface stress, we also evaluated the corresponding detectable sensing signals. We performed FEA simulations on both a cantilever and an MSS which are coated with poly (methyl methacrylate; PMMA); a commonly used polymer as a receptor material, which has the Young's modulus and the Poisson's ratio of 3 × 10^9^ Pa and 0.4, respectively. The thickness was set at their optimum values: 2.5 and 8.0 μm for the cantilever and the MSS, respectively (Yoshikawa, 2011; Yoshikawa et al., 2014). The minimum isotropic internal strain or surface stress which induces detectable signals (e.g., 1 nm deflection for the cantilever and 10^−6^ relative resistance change for the MSS) has been estimated as 10^−9^ ~ 10^−8^ or 10^−5^ ~ 10^−4^ N/m, respectively.

In terms of the receptor material, it has been confirmed that higher sensing signals can be obtained by using a material with low Young's modulus (<10^9^ Pa). This is a favorable condition for practical applications because most biological materials which can interact with viruses have a low Young's modulus. For example, the Young's moduli of proteins and cells which are typically used for viral cultures are 10^4^ ~ 10^9^ Pa and ~10^4^ Pa, respectively. Another advantage of using soft materials is that signals are less affected by thickness, leading to higher reproducibility for virus detection. It should be noted that the force caused by the viruses may not be fully transmitted to the cantilever or MSS membrane in an actual measurement system if the receptor layer is thick and soft. Since viscoelastic properties are not taken into account in the present simulations, signals can be reduced as a result of stress relaxation within a receptor layer.

As living cells are the only media where viruses can be cultivated, there is a possibility that living cells are used as receptor materials. It is expected that selectivity will be enhanced depending on the cell types only if cells are stably maintained on nanomechanical sensors. However, the noise caused by cell migration is one of the biggest experimental issues. Bischofs et al. measured the surface stress induced by an endothelial cell and estimated it to be ~2 mN/m (Bischofs et al., 2009). Our FEA simulations showed that a surface stress of 2 mN/m leads to a sensing signal comparable to adsorption of ~10^2^ viruses on a receptor layer. Thus, the cellular force should give rise to a non-negligible background noise. It is estimated that at least 10^2^~ 10^3^ of viruses are needed to obtain a detectable sensing signal when living cells are used as receptor materials.

Finally, let us compare the practical aspects between a cantilever and MSS. While the sensitivity of a cantilever sensor with an optical read-out is comparable to that of an MSS, a cantilever sensor requires a bulky instrumentation. In contrast, a compact measurement system can be realized with an MSS because of the chip-integrated piezoresistive electric sensing. Furthermore, MSS exhibits higher robustness to the adsorption position and in homogeneous coating (Loizeau et al., 2015). Thus, MSS seems to be a more favorable option for practical applications such as mobile environmental monitoring systems, point-of-care-testing (POCT) and primary screening for infectious diseases, etc. Although the sensitivity of MSS is not as high as that of enzyme-linked immunosorbent assay (ELISA), which can detect ~10^1^ of viruses (Pineda et al., 2009), MSS is a promising candidate for a portable real-time virus detection device based on its simple detection mechanism and the easier experimental protocols.

## Author contributions

GI, KS, and GY designed the simulation models. GI conducted the finite element analysis, and wrote the main paper. All authors discussed the results and implications, and commented on the manuscript at all stages.

### Conflict of interest statement

The authors declare that the research was conducted in the absence of any commercial or financial relationships that could be construed as a potential conflict of interest. The reviewer TY and handling Editor declared a current collaboration and the handling Editor states that the process nevertheless met the standards of a fair and objective review.
